# Conflicting health information: a critical research need

**DOI:** 10.1111/hex.12438

**Published:** 2015-12-28

**Authors:** Delesha M. Carpenter, Lorie L. Geryk, Annie T. Chen, Rebekah H. Nagler, Nathan F. Dieckmann, Paul K. J. Han

**Affiliations:** ^1^ Department of Pharmaceutical Outcomes and PolicyUniversity of North Carolina at Chapel HillAshevilleNCUSA; ^2^ Department of Biomedical and Health InformaticsUniversity of WashingtonSeattleWAUSA; ^3^ School of Journalism & Mass CommunicationUniversity of MinnesotaMinneapolisMNUSA; ^4^ Decision ResearchOregon Health & ScienceUniversity PortlandORUSA; ^5^ Center for Outcomes Research and EvaluationMaine Medical CenterPortlandMEUSA; ^6^ Tufts University Clinical and Translational Sciences InstituteBostonMAUSA

**Keywords:** conflicting information, decision‐making, health

## Abstract

Conflicting health information is increasing in amount and visibility, as evidenced most recently by the controversy surrounding the risks and benefits of childhood vaccinations. The mechanisms through which conflicting information affects individuals are poorly understood; thus, we are unprepared to help people process conflicting information when making important health decisions. In this viewpoint article, we describe this problem, summarize insights from the existing literature on the prevalence and effects of conflicting health information, and identify important knowledge gaps. We propose a working definition of conflicting health information and describe a conceptual typology to guide future research in this area. The typology classifies conflicting information according to four fundamental dimensions: the substantive issue under conflict, the number of conflicting sources (multiplicity), the degree of evidence heterogeneity and the degree of temporal inconsistency.

Conflicting health information is a growing problem worldwide, as evidenced by high‐profile controversies surrounding childhood vaccination and numerous other health issues. Mass media have increased the visibility of such conflicting and often politically charged controversial health information,^1^ while a growing professional emphasis on involving individuals in health‐care decisions has increased the exposure of patients and providers to conflicting health information in clinical encounters.^2^ More than ever before, patients, providers, caregivers and policy makers are expected to evaluate conflicting health information from different sources, judge whether the information is credible and decide how to respond.

Helping people process and evaluate conflicting health information is an increasingly important need in health care, yet for several reasons, this need remains largely unmet. Studies of the prevalence, causes and effects of conflicting health information have been limited. Although behavioural research outside of the health‐care domain has yielded insights on how individuals process and evaluate conflicting information, these insights have not been translated to health care. Thus, evidence‐based strategies to help patients make sense of conflicting health information are lacking. The purpose of this viewpoint article was to highlight what is currently known about the prevalence, causes and effects of conflicting health information and to outline an organized approach for future research aimed at addressing current knowledge gaps.

## Conflicting health information: prevalence, causes and effects

Evidence on the prevalence of conflicting health information is limited to the findings of a small number of studies that examine the perceptions of patients, physicians and the general public regarding health topics such as medications, cancer screening and nutrition. Studies suggest that 18–80% of patients receive conflicting medication information,^3^, ^4^, ^5^, ^6^, ^7^ while approximately 50–75% of patients^8^, ^9^, ^10^ and providers^11^ perceive conflicting information about cancer‐screening guidelines. Additionally, 72% of US adults report medium to high exposure to conflicting nutrition information.^12^

Evidence on the causes or sources of conflicting health information is also limited, although studies have begun to shed light on these issues. People may encounter conflicting information actively, while searching for health information,^13^ or passively, as recipients of unsolicited health advice.^14^ Conflicting health information may originate from professional and lay sources including the Internet, written materials^15^ and personal testimonials.^4^ One study that examined 15 different information sources found that physicians, media and the Internet were the most common patient‐reported sources of conflicting medication information.^4^ The Internet^16^, ^17^, ^18^ and media^12^, ^19^, ^20^ in particular present a plethora of conflicting information on numerous health topics,^16^, ^17^ from screening guidelines to vaccinations. Social media is an increasingly common forum for dialogue about health issues, and studying exposure to conflicting health information on social media and in other informal settings is increasingly important given the growing number of individuals obtaining heath information through such channels.^19^

Conflicting health information also has several potential negative effects, perhaps the most important of which is confusion among patients and providers. When people encounter conflicting health information, they may have trouble deciding whom to trust and may defer to the source they deem most credible.^20^ Although conflicting expert opinions about complex issues such as health‐care problems are arguably natural and expected, laypersons may perceive such conflict as evidence of intentional bias or expert incompetence.^21^ These beliefs, in turn, have been associated with lower intentions to engage in health behaviours for which there is clear scientific consensus (e.g. fruit/vegetable consumption).^12^ Empirical evidence suggests that conflicting information may also increase anxiety,^22^ heighten risk perceptions,^23^ decrease the ability of individuals to assess the reliability of information sources^24^ and reduce medication adherence.^3^, ^4^

When people encounter conflicting health information, they may also try to make sense of it using various strategies, including filtering out misinformation,^25^ seeking additional information from a health‐care provider^14^ and developing more sophisticated strategies to appraise it.^26^ Because greater cognitive effort is required to process contradictory vs. congruent information, conflicting health information may increase individuals’ use of heuristics – or mental shortcuts – that may exacerbate cognitive biases or lead to errors in judgment.^27^ For example, they may only focus on one source of information, such as their doctor,^28^ and leave out other important elements,^29^ or attend to and use information that is easier to evaluate.^30^, ^31^ In addition, the heightened uncertainty that arises from conflicting information may motivate people to choose information sources and interpretations that are most consistent with what they want to believe.^32^, ^33^ Experimental work by Brewer and colleagues found that discordant genetic and standard test results for risk of recurrence of breast cancer did not significantly change women's preferences for chemotherapy.^34^ Conflicting information may also result in decision paralysis – leading people to simply do nothing at all (a response known as the status quo bias).^35^

## Conflicting health information: a working definition

Past research suggests that conflicting health information is common, increasing in volume and visibility, and deleterious in its psychological and behavioural effects; however, the existing evidence base on this problem is limited. The critical need moving forward is to address knowledge gaps on the prevalence, causes and effects of conflicting information, but a prerequisite for such research is conceptual clarity on how conflicting health information should be defined.

We propose defining conflicting health information in terms of health‐related propositions. By ‘propositions’ we mean statements or assertions about a health‐related issue. These propositions, furthermore, may pertain to health‐related scientific evidence, interpretations of the evidence or recommendations and guidelines issued by experts or other individuals. Propositions may originate from either a single source or multiple information sources and may either be actively sought by an individual (e.g. through an Internet search to determine whether coffee is bad for your heart) or passively encountered (e.g. through an overheard conversation about how coffee causes heart disease).

‘Conflicting health information’ can then be operationally defined as two or more health‐related propositions that are logically inconsistent with one another. The Merriam‐Webster dictionary defines the term ‘conflicting’ as ‘being in conflict, collision, or opposition’ or ‘incompatible’.^36^ The defining feature of ‘conflicting’ in our definition is that the propositions are discrepant such that a person could not simultaneously engage in or believe both propositions at once. For example, if two propositions differed on one point, such as the recommended age to initiate mammography screening (age 40 or 50), a woman could not initiate screening at both age 40 or 50. Similarly, if a person found a proposition online that ‘coffee is bad for your heart’ and the person's physician told him/her that ‘coffee is not bad for your heart’, the person could not simultaneously believe both propositions.

## Conflicting health information: a provisional conceptual typology

Beyond defining the meaning of the phenomenon, conflicting health information can be classified according to four fundamental dimensions: the substantive issue under conflict, the number of conflicting sources (multiplicity), the degree of evidence heterogeneity and the degree of temporal inconsistency. In Table 1, we present text excerpts of conflicting propositions related to measles–mumps–rubella (MMR) vaccinations to illustrate these four dimensions.

**Table 1:** Conflicting health information typology applied to measles–mumps–rubella (MMR) vaccinations

Dimension	Definition	Case	Examples of Conflicting Information for the Specific Proposition: ‘I should get my child vaccinated’
Issue of conflict	The specific health topic (e.g. diagnosis, prognosis, cause, treatment) for which conflicting information exists	Vaccine schedule vs. vaccine risk	–
Multiplicity	The number of sources that provides conflicting information about a health issue		
	*Single source*: conflicting information exists within the same source	An Amazon discussion forum includes conflicting information threads.	Amazon discussion forum:^a^ On 17 July 2010, 8:42:11 PM PDT, bens_mom says: ‘For those of you who still believe the MMR vaccine is the cause of autism, please read this article. You will find that the study linking the MMR vaccine to autism was not only sponsored by a party who wanted to link the vaccine to autism, but the doctor (Dr. Wakefield) who performed the study stood to gain a lot from an outcome where the vaccine was proven unsafe’. On 23 July 2010, 7:47:35 AM PDT, Hot hands says: ‘bla bla bla….explain to me, then, how children who are normal and healthy go down the tubes….right after receiving (sic) a vaccine? Thousands? (hair splitter) of parents, who witness this themselves, cannot be wrong…sorry’
	*Multiple sources*: conflicting information exists across two or more sources	A government agency and a physician make different recommendations about the MMR vaccination.	Recommendations of the ACIP:^51^ ‘For the prevention of measles, mumps, and rubella, vaccination is recommended for persons aged ≥12 months’ Recommendation from Dr. Tenpenny:^b^ ‘The decision to vaccinate or not is important and complex. Parents must take on the responsibility to seek enough information to make an educated, informed decision’
Evidence heterogeneity	The extent to which the conflicting information comes from similar or differing evidentiary types		
	*Homogenous:* conflicting information from similar evidentiary types	Two journal articles report different findings about the link between MMR vaccinations and autism.	The Lancet (1998):^38^ ‘…the onset of [regressive developmental disorder] behavioural problems had been linked, either by the parents or by the child's physician, with measles, mumps, and rubella vaccination’ The Lancet (1999):^52^ ‘Our analyses do not support a causal association between MMR vaccine and autism’
	*Heterogeneous* **:** conflicting information from differing evidentiary types	Conflicting vaccination information from a tabloid publication and a journal article	Daily Mail (tabloid publication) (2006)^53^: ‘New American research shows that there could be a link between the controversial MMR triple vaccine and autism and bowel disease in children’ American Journal of Public Health (journal article) (2004):^54^ ‘On the basis of the results of this review, the GACVS agreed and concluded that there is no evidence for a causal association between MMR vaccine and autism or autistic spectrum disorders’
Temporal inconsistency	Whether conflicting propositions about an issue exist at the same point in time or at different points in time		
	*Synchronous*: Conflicting propositions that exist at the same point in time	Current divergence in the existing clinical practice guidelines on the vaccination schedule issued by the ACIP and NACI	Recommendations of the ACIP:^51^ ‘Currently, ACIP recommends 2 doses of MMR vaccine routinely for children with the first dose administered at age 12 through 15 months and the second dose administered at age 4 through 6 years before school entry’ Recommendations of the NACI:^55^ ‘NACI recommends that the first dose of MMRV be administered at 12–15 months of age, and the second at 18 months or at 4–6 years of age (preschool)’
	*Asynchronous*: Conflict between older and newer propositions	The 1989 ACIP statement compared to the 1987 ACIP statements	29 December 1989:^56^ The ACIP revised recommendations on measles prevention to include a two‐dose schedule using combined MMR vaccine 1987: The ACIP recommendation included a one‐dose schedule

MMR, measles–mumps–rubella; ACIP, Advisory Committee on Immunization Practices; NACI, National Advisory committee on Immunization; GACVS, Global Advisory Committee on Vaccine Safety.

a Amazon discussion forum. MMR vaccine DOES NOT cause autism!!!. July 2010. Available at: http://www.amazon.com/forum/parenting?_encoding=UTF8&cdForum=Fx20C498EK5JY4S&cdThread=Tx9PZWGZL9NYYJ, accessed 10 February 2015.

b Drtenpenny.com. When Parents Question Vaccination [Blog post]. 2013 [cited 2015]. Available at: http://drtenpenny.com/when-parents-question-vaccination/.

The first dimension of conflicting health information is the *issue of conflict* or specific health topic for which conflicting information exists. Issue of conflict is important because people's reactions to conflicting information may vary based on the topic. For example, people may have more negative reactions to conflicting information regarding vaccine risk than the schedule for receiving vaccinations. The second dimension is *multiplicity*, or the number of different sources of conflicting information. This dimension is potentially important because the sheer number of sources of conflicting information may moderate its effects. For example, people may react differently if they encounter conflicting information about vaccines from a single source vs. multiple sources, and people's negative reactions may increase or plateau as the number of conflicting sources increases. *Evidence heterogeneity*, the third dimension, is important because individuals may process conflicting information differently when it comes from homogenous sources, such as scientific studies, vs. heterogeneous sources, such as scientific studies and personal anecdotes. Using the vaccine example, a clinician may react differently to conflicting vaccine safety information from two scientific journals than to conflicting information from a scientific journal and a patient testimonial. *Temporal inconsistency*, the temporal relationship between conflicting propositions, is the final dimension. Conflicting information that is asynchronous (reflecting inconsistency between logical propositions separated by time) may produce different behavioural effects than conflicting information that is synchronous (reflecting inconsistency between logical propositions that exist simultaneously). Importantly, this problem is common and inherent to the normal advancement of scientific knowledge – a process in which lower quality evidence (e.g. from observational studies) is often supplanted by higher quality evidence [e.g. from randomized controlled trials (RCTs)]. An additional consideration for asynchronous conflicting information is the frequency with which conflicts arise; if there is less time between exposures to conflicting propositions, this may have a more negative impact on individuals than if there is a greater period of time between exposures to conflicting propositions. For example, if MMR guidelines change yearly, this may more negatively impact trust in sources than if the guidelines change every 5 years.

Our proposed conceptual typology focuses on properties of conflicting information itself; according to our definition, health‐related propositions are either conflicting or not. However, we recognize that *perceptions* of conflicting information, rather than the objective existence of conflicting information, are important determinants of people's behavioural responses. These perceptions, in turn, are determined by numerous factors including the complexity of information. For example, individuals may easily recognize conflicting health propositions that directly oppose each other: ‘coffee is good for your heart’ and ‘coffee is bad for your heart’. In contrast, individuals may find it difficult to identify conflict in the propositions ‘coffee is bad for your heart’ and ‘coffee is bad for your heart if you drink more than one cup per day’. Additionally, the personal salience of information may influence the degree to which individuals perceive informational conflict. Using the coffee example, someone who does not drink coffee may be less likely to recognize conflicting propositions about coffee.

Several other factors may cause individuals to perceive informational conflict when it does not exist. For example, a person may perceive the absence of information as conflicting information if he/she experiences a rare medication side‐effect of which he/she was never informed. Individuals may also perceive informational conflict when confronted by logically consistent propositions that have different health implications. For example, a person exposed to the propositions ‘coffee is bad for your heart’ and ‘coffee prevents Type II diabetes’ may perceive a conflict about *whether to drink coffee,* given that coffee drinking has both positive and negative health outcomes. However, such conflict is decisional rather than informational, because the propositions themselves are not logically inconsistent. Our operational definition focuses on informational conflict resulting from logical inconsistency in alternative health propositions, rather than decisional conflict resulting from competing pros and cons of alternative choice options. Therefore, although the coffee and diabetes example presented above would not constitute an instance of informational conflict, the causes and mechanisms underlying individuals’ subjective perceptions of conflict and how subjective perceptions affect health decisions are important areas for future research. Apparent conflicts in information may also result from differences in the particular outcomes that are reported in the coverage of research studies. For example, one news story may focus on a surrogate outcome, such as prevention of heart attacks, and report a significant benefit of a drug, while other reports may focus on cardiovascular disease mortality and report no benefit. Although these reporting differences may reflect expert disagreements over which outcomes ought to be publicized, they do not reflect true conflicts in the information *per se*. Finally, the nature of the decision at hand as well as the background of the individual making the decision may determine the extent to which a person perceives informational conflict. For example, a patient who must make a decision for which conflicting information exists – for example to undergo prostate cancer screening with the prostate‐specific antigen test – may perceive informational conflict in scientific knowledge, while a researcher (whose goal is to simply produce and evaluate the existing knowledge) may simply perceive available scientific evidence as being of limited quantity and/or heterogeneous quality, and not necessarily ‘conflicting’.

At times, informational conflict may also reflect the varying strength of scientific evidence and thus may be more perceived than real. Observational and experimental studies may yield seemingly conflicting results that can take years of research and additional studies to reconcile. Individuals trained in research methodology understand that observational studies yield weaker evidence – which needs to be viewed sceptically – while RCTs yield stronger evidence that can invalidate weaker evidence. Thus, for a scientifically trained audience, apparent inconsistencies in the results of observational and RCT findings may not necessarily be perceived as ‘conflicting information’. Limited scientific literacy, however, may prevent the general public from understanding the hierarchy of evidence and the differences between correlational and causal studies; as a result, they may simply perceive such inconsistencies in evidence as conflicts between equivalent forms of evidence. For example, the public may perceive conflict between a cross‐sectional survey of 50 000 people that finds that coffee is associated with a lower risk of heart disease and an RCT with 2500 participants that finds no association. In contrast, nutritional epidemiologists would regard these studies as non‐comparable from an evidentiary standpoint and prioritize the RCT findings. Failure to understand the hierarchy of scientific evidence may also explain why attempts to educate the public about RCTs that found no evidence of a vaccination–autism link have not made much headway in diffusing the negative effects of conflicting information.^37^

Fraudulent scientific studies can also produce conflicting information – and perpetuate perceptions of conflict – among professional communities as well as the general public. The vaccination example given in Table 1 offers an excellent example of this. In 1998, Wakefield and colleagues published a paper in which they claimed that environmental triggers (i.e. the MMR vaccination) were associated with gastrointestinal disease and developmental regression (i.e. autism) in eight of 12 children studied.^38^ In 2010, twelve years later, the paper was officially retracted due to ethical misconduct and falsified data.^39^ In 2011, detailed information regarding why the findings on the link between autism and MMR vaccination were fraudulent was published in *BMJ*.^40^ Unfortunately, during the 12 years before the paper was retracted, the findings were disseminated in media and online outlets and served as a basis for the modern antivaccination movement.^41^ In fact, one study found that half of antivaccination websites applaud doctors such as Wakefield for speaking out against vaccinations.^41^ Despite repeated and large‐scale efforts to educate the general public about why MMR vaccination does not cause autism, the antivaccination movement remains strong and continues to cite Wakefield's retracted study.

## Future research directions

Many gaps exist in our understanding of conflicting health information. Thus, we are not well‐positioned to help patients and clinicians manage this growing problem. Moving forward, we believe a coherent, comprehensive programme of research is needed to determine the prevalence, causes and effects of conflicting health information and to develop effective strategies to deal with it. The multifaceted nature of this phenomenon and its potential effects calls for a transdisciplinary programme of research. However, both the challenge and promise of employing a transdisciplinary team of scientists (e.g. communication scholars, behavioural decision theorists, health services researchers) are that they have their own well‐developed theories of how people process information and make decisions. These theories may provide useful lenses for understanding the causes and effects of conflicting information; however, exactly how to optimally integrate these theories is unclear and represents a fundamental research need. For example, theories from the persuasion literature, including extended parallel process model^42^ and the unimodel^43^, ^44^, may offer guidance as to how subjective perceptions (e.g. perceived threat) may influence processing of conflicting health information. Additionally, other theories, such as fuzzy trace theory,^45^ are applicable to the study of conflicting health information because they identify factors (e.g. low health literacy) that may interfere with information processing. Finally, theosries from other fields, such as artificial intelligence, offer potentially useful insights on how people process and integrate conflicting information.^46^, ^47^, ^48^

Equally important is the need to develop valid and reliable measures of conflicting health information. Some measures exist,^4^, ^49^, ^50^ but more work is needed to assess their psychometric properties and to develop measures that accurately capture conflicting health information in its many potential manifestations. This initial psychometric work could then enable basic research aimed at elucidating the effects of conflicting health information on health judgments and decisions and the mechanisms underlying these effects. Additional measurement work should be devoted to developing instruments that capture the intrapersonal factors (e.g. beliefs, information needs, perceived salience of topic) that are likely associated with how individuals process conflicting information.

Understanding the effects of conflicting health information would ultimately pave the way for applied research aimed at translating insights on the causes and effects of conflicting health information into interventions to help individuals better manage this information. We have proposed several key issues for consideration; however, there are additional issues related to study design and data analysis that could perpetuate conflicting information. For example, a single study could result in conflicting information if researchers use alternative statistical methodologies that result in different interpretations of the data. The fundamental prerequisite for a comprehensive programme of research on conflicting health information, however, is recognition of the phenomenon's importance and conceptual clarity about what it entails. This viewpoint article is offered as a preliminary step towards these goals.

## Sources of funding

Financial support for this research was provided in part by grants from the National Institutes of Health. The funding agreement ensured the authors’ independence in designing the research, interpreting the data, writing and publishing the report.

## Conflict of interests

The authors have no conflict of interests to disclose.
